# Enhanced risk prediction of femoral head osteonecrosis in the elderly: a comparative study of random forest and logistic regression models

**DOI:** 10.3389/fmed.2025.1640085

**Published:** 2025-12-10

**Authors:** Peng Shang, QingQing Liu, Hao Mu, Haijin Yang, Junqing Jia

**Affiliations:** Department of Orthopedics, Shanxi Bethune Hospital, Tongji Shanxi Hospital, Shanxi Academy of Medical Sciences, Third Hospital of Shanxi Medical University, Taiyuan, China

**Keywords:** avascular necrosis, femoral head, elderly, random forest, logistic regression, prediction model, risk factors

## Abstract

**Background and aim:**

Osteonecrosis of the femoral head (ONFH) is a degenerative joint disorder that frequently leads to structural collapse and impaired mobility, particularly in older adults. Early detection of associated risk factors is essential for timely intervention. This study aimed to compare the predictive performance of a Random Forest (RF) algorithm and a Logistic Regression (LR) model in identifying key contributors to ONFH in elderly patients.

**Methods:**

This retrospective study included 339 patients aged ≥ 75 years who received treatment at Shanxi Bethune Hospital from January 2017 to December 2023, with complete clinical and imaging records. Variables included demographics, bone mineral density, medication and lifestyle history, comorbidities, and radiographic findings. Patients were randomly allocated into training (70%) and validation (30%) cohorts. Predictive models were developed using RF and LR, with performance assessed by accuracy, sensitivity, specificity, and area under the receiver operating characteristic curve (AUC).

**Results:**

Both models consistently identified corticosteroid exposure, reduced bone mineral density, prior femoral fractures, and advanced age as major risk factors. The RF model demonstrated superior performance (AUC = 0.896; accuracy = 83.5%; sensitivity = 82.4%; specificity = 84.3%) compared to the LR model (AUC = 0.797; accuracy = 75.0%; sensitivity = 72.0%; specificity = 76.0%). ROC analysis confirmed the RF model’s enhanced discriminative ability.

**Conclusion:**

The RF algorithm outperformed traditional logistic regression in predicting ONFH among older adults, highlighting the potential of machine learning techniques to support early risk identification and improve clinical decision-making in orthopedic care.

## Introduction

Avascular necrosis of the femoral head (AVNFH), or osteonecrosis, is a progressive condition characterized by impaired blood flow to the femoral head, resulting in bone tissue death, structural weakening, and eventual collapse of the joint surface (1–3). Without timely intervention, AVNFH can result in severe hip dysfunction, chronic pain, and the eventual need for total hip arthroplasty (THA). While AVNFH may occur in younger populations due to trauma or corticosteroid use, the disease burden is particularly profound among elderly patients, who often suffer from age-related degenerative changes, vascular insufficiency, and systemic comorbidities (4).

In older adults, AVNFH progression is frequently insidious and underdiagnosed until advanced stages, at which point treatment options become limited. Age-related factors such as impaired bone remodeling capacity, reduced vascular elasticity, and high prevalence of osteopenia or osteoporosis further exacerbate femoral head vulnerability. Moreover, many elderly patients are exposed to modifiable risk factors such as long-term glucocorticoid therapy for chronic diseases, alcohol consumption, and metabolic syndrome, all of which have been strongly implicated in the pathogenesis of AVNFH (5–7). These factors contribute to a rising incidence of non-traumatic AVNFH among aging populations worldwide. From a clinical standpoint, accurate prediction of AVNFH risk in the elderly is critical for several reasons. First, early-stage AVNFH is often asymptomatic and may be missed in routine orthopedic screening. Second, elderly individuals with limited mobility may not report early symptoms, delaying diagnosis and missing the window for joint-preserving interventions such as core decompression or pharmacologic treatment. Third, late-stage AVNFH in this population often necessitates joint replacement surgery, which is associated with higher perioperative risks, longer recovery times, and increased healthcare costs. Therefore, early risk stratification could play a vital role in improving outcomes and guiding individualized management strategies (8–10).

Despite its clinical importance, there is a lack of robust, validated predictive models tailored specifically to the elderly population for AVNFH. Traditional statistical models such as logistic regression, while useful in identifying linear associations, often fail to capture complex, nonlinear interactions among multiple risk variables, limiting their predictive accuracy. This is particularly problematic in elderly patients, where comorbidity clusters and multifactorial etiologies are common. Recent advancements in machine learning (ML) offer promising solutions to these limitations. Among them, the Random Forest (RF) algorithm has gained recognition for its ability to handle high-dimensional data, detect variable interactions, and provide robust predictive performance across diverse medical domains. RF models are ensemble-based, resistant to overfitting, and inherently capable of ranking variable importance, making them ideal tools for risk modeling in heterogeneous clinical populations (11, 12).

This study aims to construct and compare the performance of Random Forest and Logistic Regression models in predicting the risk of AVNFH among elderly patients based on retrospective clinical data. By integrating demographic, clinical, imaging, and treatment-related variables, we seek to develop a reliable, interpretable prediction model that can assist clinicians in early identification of high-risk individuals, support preventive strategies, and ultimately enhance quality of life in geriatric orthopedic care.

## Materials and methods

### Study design and data collection

This retrospective study was conducted on 339 elderly patients aged 75 years or older who were admitted to the Department of Orthopedics at Shanxi Bethune Hospital between January 2017 and December 2023. Eligible patients had complete clinical records, hip joint imaging data (X-ray or MRI), and at least 1 year of radiologic follow-up. Baseline data were collected at the initial hospital visit or diagnosis. The outcome variable was the presence or absence of avascular necrosis of the femoral head (AVNFH), as assessed during follow-up imaging.

The dataset included detailed demographic characteristics, clinical features, comorbidities, laboratory indicators, medication history, and imaging findings. Data were anonymized before analysis. This study was approved by the Ethics Committee of Shanxi Bethune Hospital (No. 151735187) and was conducted in accordance with the Declaration of Helsinki.

### Inclusion and exclusion criteria

Inclusion criteria consisted of age 75 years or above, availability of complete baseline demographic and clinical information, and at least 1 year of follow-up imaging suitable for AVNFH assessment. Exclusion criteria included traumatic femoral head necrosis (e.g., secondary to hip fracture), active malignancy with bone metastasis, hip joint infection or inflammatory arthritis (e.g., SLE), osteoarthritis, or missing key clinical or imaging data. These conditions were excluded to avoid confounding factors that could influence the risk of AVNFH.

### Baseline data collected

Baseline data collected at the initial visit included demographic information (age, sex), BMI, medical history (hypertension, diabetes, osteoporosis), medication history (corticosteroid use), and lifestyle factors (smoking, alcohol use). Additionally, bone mineral density (BMD), femoral head imaging (X-ray or MRI), and comorbid conditions were assessed to identify potential risk factors for AVNFH. Missing data were handled using multiple imputation if the proportion of missing data was less than 5%. For variables with more than 10% missing data, patients with critical missing values were excluded from analysis. Hyperparameter tuning for the Random Forest model was conducted using grid search, with key parameters including the number of trees (n_estimators = 100), maximum depth (max_depth = 10), and minimum samples per leaf (min_samples_leaf = 5).

### Definition of AVNFH

AVNFH was defined radiologically based on one or more of the following features: collapse or flattening of the femoral head, the presence of the crescent sign, or characteristic bone marrow changes visible on MRI. MRI diagnostic criteria included focal hypointensity on T1-weighted images and a “double-line sign” on T2-weighted sequences. AVNFH was staged based on radiological features, including the collapse or flattening of the femoral head, the presence of the crescent sign, and characteristic bone marrow changes visible on MRI. MRI staging criteria included focal hypointensity on T1-weighted images and a “double-line sign” on T2-weighted sequences. Radiological assessments were conducted independently by two senior musculoskeletal radiologists. Discrepancies were resolved through joint review.

### Variable selection and logistic regression modeling

All potential predictors were first evaluated using univariate logistic regression. Variables with a *p* < 0.1 were entered into the multivariate model. To achieve a more parsimonious model, backward stepwise selection was performed based on the Akaike Information Criterion (AIC). Multicollinearity was tested using variance inflation factor (VIF), with only variables having VIF values below 10 included in the final model. Interaction terms were explored but not included due to a lack of statistical significance.

### Dataset splitting

Continuous variables (e.g., age, BMI, and bone mineral density) were standardized prior to analysis. Feature selection combined traditional statistical methods—chi-squared tests for categorical variables and ANOVA for continuous ones—with feature importance scores from the Random Forest model.

To address class imbalance between patients with and without avascular necrosis of the femoral head (AVNFH), we applied the Synthetic Minority Oversampling Technique (SMOTE) to the training cohort. In the original dataset, 88 patients (25.9%) were diagnosed with AVNFH, while 251 patients (74.1%) were controls, resulting in an approximate imbalance ratio of 1:3. SMOTE was implemented exclusively on the training dataset (*n* = 237) to generate synthetic minority class samples and prevent data leakage. The validation dataset (*n* = 102) remained unaltered to preserve its real-world distribution and enable an unbiased assessment of model generalizability. For feature selection, we first conducted univariate logistic regression analyses, retaining variables with a *p* < 0.05. These candidate variables were subsequently included in the multivariate logistic regression model and random forest model for predictive modeling.

### Dataset splitting and cross-validation

The dataset was randomly split into a training set (70%, *n* = 237) and a validation set (30%, *n* = 102). Within the training set, five-fold cross-validation was used to assess model stability and tune hyperparameters. Final models were then retrained on the full training set using the optimized parameters and evaluated on the validation set to assess independent performance.

### Model development

Two predictive models were constructed for comparison: a multivariate logistic regression and a Random Forest classifier. Logistic regression served as a conventional baseline for modeling linear associations between predictors and the binary outcome of AVNFH. In contrast, the Random Forest algorithm—a tree-based ensemble technique—was employed to capture complex nonlinear patterns and enhance predictive accuracy. Hyperparameter optimization for the Random Forest model was conducted via grid search, considering parameters such as the number of trees (n_estimators), maximum depth (max_depth), minimum samples to split a node (min_samples_split), and minimum samples per leaf (min_samples_leaf). Model performance was evaluated using the area under the ROC curve (AUC), with preference given to configurations achieving the highest AUC and lowest out-of-bag (OOB) error during cross-validation. In addition to accuracy, sensitivity, specificity, and AUC, we also evaluated precision, recall, and the F1-score for both models. These metrics provide a more comprehensive view of model performance, particularly in the context of imbalanced datasets. Given the imbalance in the dataset, with 25.9% of patients diagnosed with AVNFH and 74.1% as controls, we applied the Synthetic Minority Over-sampling Technique (SMOTE) to the training cohort to balance the classes. Categorical variables were encoded using one-hot encoding or label encoding as appropriate for model training.

### Model evaluation and feature importance analysis

After training, both models were tested on the independent validation set. Performance was evaluated using four key metrics: accuracy, sensitivity, specificity, and AUC-ROC. The predictive performance of both models was compared using ROC curves. To further evaluate the clinical utility of the models, we performed Decision Curve Analysis (DCA) for both the logistic regression and Random Forest models. DCA was applied across a range of threshold probabilities, and the net benefits were compared to a scenario of no intervention and a scenario of universal intervention. The results indicate that both models provided a net benefit over the entire range of threshold probabilities, with the Random Forest model demonstrating higher net benefit compared to the logistic regression model in both the training and validation cohorts. Additionally, the Random Forest model provided a ranked list of feature importances, indicating the relative influence of each predictor variable on model output. These results were used to interpret the clinical significance of key predictors in AVNFH risk assessment.

### Primary and secondary outcomes

The primary outcome was the presence or absence of AVNFH, as determined by follow-up imaging. Secondary outcomes included the predictive performance metrics (accuracy, sensitivity, specificity, and AUC) of the Logistic Regression and Random Forest models in identifying key risk factors for AVNFH

## Data analysis

All analyses were conducted using Python (version 3.10), with key libraries including Scikit-learn, Pandas, NumPy, Matplotlib, and Seaborn. Descriptive statistics summarized baseline characteristics: continuous variables were reported as mean ± standard deviation (SD) or median with interquartile range (IQR), depending on normality, and compared using Student’s *t*-test or Mann–Whitney U test. Categorical variables were presented as counts and percentages and analyzed using the chi-square test or Fisher’s exact test, as appropriate.

Model development followed a two-phase approach. Initially, logistic regression and Random Forest models were trained on the training dataset, with five-fold cross-validation employed for hyperparameter tuning and internal validation. Subsequently, the optimized models were tested on the independent validation dataset. Predictive performance was assessed using accuracy, sensitivity, specificity, and the area under the ROC curve (AUC-ROC).

In the logistic regression model, the statistical significance of each predictor was evaluated by calculating odds ratios (ORs) with corresponding 95% confidence intervals (CIs). A *p* < 0.05 was considered indicative of statistical significance. For the Random Forest model, feature importance scores were derived from the Gini impurity index and visualized to aid in interpretation.

All plots and visual summaries—including ROC curves, confusion matrices, and variable importance charts—were generated using Matplotlib and Seaborn libraries. Missing data were handled using multiple imputation if the proportion was less than 10%; otherwise, patients with critical missing values were excluded from analysis. No assumptions of data normality or homoscedasticity were made for machine learning models.

## Results

### Baseline characteristics of elderly patients with and without AVNFH

**FIGURE 1:**
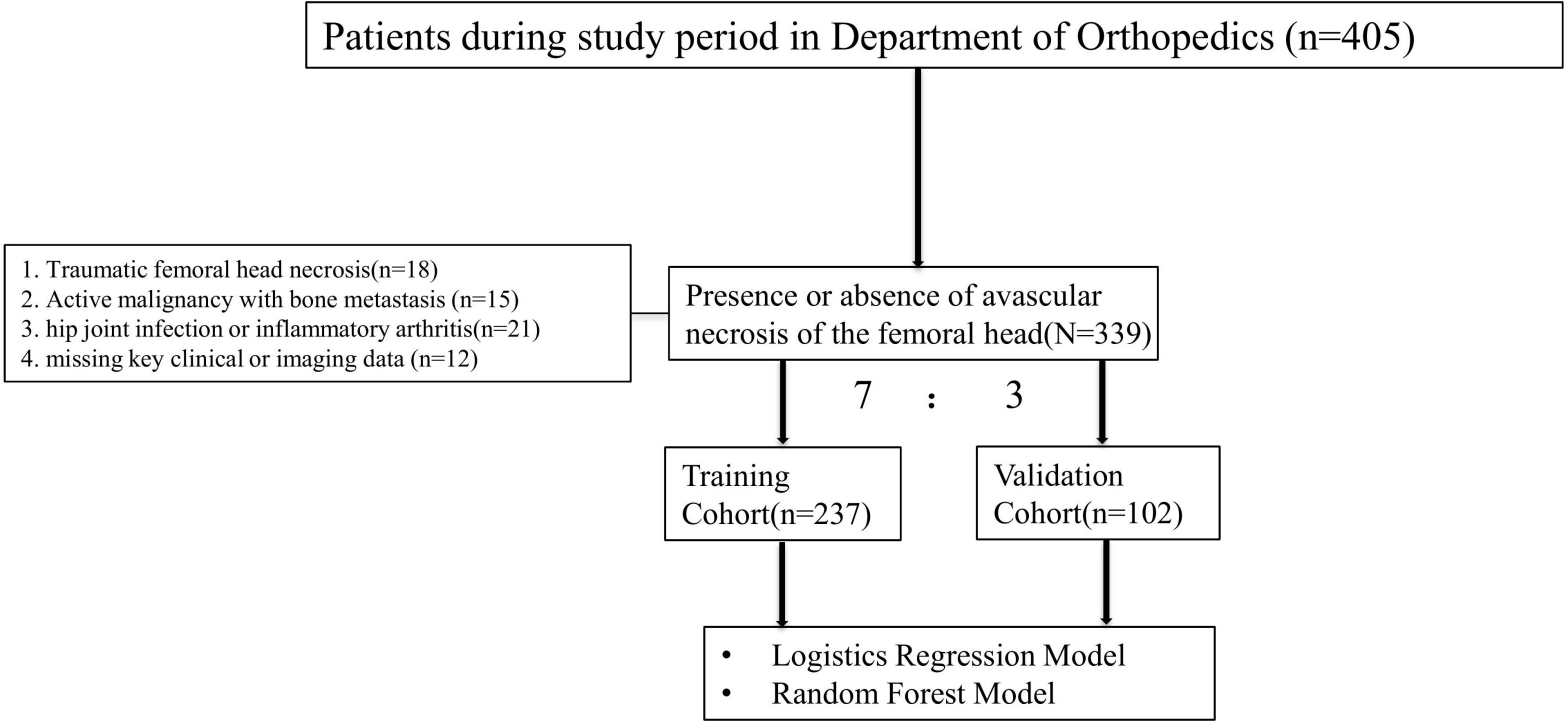
Flow diagram of patient selection, dataset division (7:3), and model development.

A total of 339 elderly patients were included in this study. The dataset was randomly divided into a training cohort comprising 237 patients (70%) and a validation cohort comprising 102 patients (30%). In the training cohort, 108 patients were diagnosed with avascular necrosis of the femoral head (AVNFH), while 129 patients had no evidence of AVNFH based on imaging and clinical criteria. In the validation cohort, 48 patients were AVNFH-positive, and 54 patients were AVNFH-negative (Figure 1).

Demographic and clinical variables were balanced between the training and validation sets, with no statistically significant differences in age distribution, sex, BMI, comorbidity profiles (including hypertension, diabetes, osteoporosis), or medication history (*p* > 0.05 for all comparisons). Bone mineral density, corticosteroid exposure history, smoking and alcohol use were also similar between the two cohorts. These findings indicate that the randomization process resulted in comparable groups suitable for model development and external validation. The detailed baseline characteristics of both cohorts are presented in Table 1.

**TABLE 1:** Baseline characteristics of patients with avascular necrosis of the femoral head in the training and validation cohorts (*n* = 339).

Variable	Training cohort (*n* = 237)	Validation cohort (*n* = 102)	*P*-value
Age (%)			0.884
<75 years	132 (55.7)	58 (56.9)	
≥75 years	105 (44.3)	44 (43.1)	
Gender (%)			0.918
Male	121 (51.1)	52 (51.0)	
Female	116 (48.9)	50 (49.0)	
BMI (%)			0.802
< 25	159 (67.1)	68 (66.7)	
≥ 25	78 (32.9)	34 (33.3)	
Corticosteroid use (%)			0.774
No	150 (63.3)	63 (61.8)	
Yes	87 (36.7)	39 (38.2)	
Smoking history (%)			0.867
No	163 (68.8)	70 (68.6)	
Yes	74 (31.2)	32 (31.4)	
Alcohol use (%)			0.931
No	172 (72.6)	74 (72.5)	
Yes	65 (27.4)	28 (27.5)	
Bone mineral density (%)			0.859
Normal	83 (35.0)	35 (34.3)	
Low (osteopenia/osteoporosis)	154 (65.0)	67 (65.7)	
Hypertension (%)			0.692
No	126 (53.2)	53 (52.0)	
Yes	111 (46.8)	49 (48.0)	
Diabetes mellitus (%)			0.739
No	160 (67.5)	70 (68.6)	
Yes	77 (32.5)	32 (31.4)	
Osteoporosis (%)			0.648
No	107 (45.1)	46 (45.1)	
Yes	130 (54.9)	56 (54.9)	
Femoral fracture history (%)			0.881
No	180 (75.9)	77 (75.5)	
Yes	57 (24.1)	25 (24.5)	
MRI signal abnormality (%)			0.812
No	157 (66.2)	66 (64.7)	
Yes	80 (33.8)	36 (35.3)	
Total cholesterol > 5.2 mmol/L (%)			0.678
No	182 (76.8)	79 (77.5)	
Yes	55 (23.2)	23 (22.5)	
LDL-C > 3.4 mmol/L (%)			0.744
No	169 (71.3)	74 (72.5)	
Yes	68 (28.7)	28 (27.5)	
Triglycerides > 1.7 mmol/L (%)			0.692
No	148 (62.4)	62 (60.8)	
Yes	89 (37.6)	40 (39.2)	
Family history of AVNFH (%)			0.866
No	213 (89.9)	91 (89.2)	
Yes	24 (10.1)	11 (10.8)	
Vitamin D deficiency (%)			0.913
No	132 (55.7)	57 (55.9)	
Yes	105 (44.3)	45 (44.1)	
AVNFH outcome (%)			–
No	129 (54.4)	54 (52.9)	
Yes	108 (45.6)	48 (47.1)	

BMI, Body Mass Index; BMD, Bone Mineral Density; LDL-C, Low-Density Lipoprotein Cholesterol; AVNFH, Avascular Necrosis of the Femoral Head. Corticosteroid use defined as systemic use for > 3 months in the previous year. *P*-values derived using chi-square test or *t*-test where appropriate.

### Univariate and multivariate logistic regression analysis

In the univariate analysis, several factors were significantly associated with AVNFH. These included long-term corticosteroid use (OR = 2.01, 95% CI: 1.41–2.86, *p* < 0.001), low bone mineral density (OR = 1.76, 95% CI: 1.24–2.51, *p* = 0.002), age ≥ 75 years (OR = 1.58, 95% CI: 1.08–2.31, *p* = 0.018), history of femoral fracture (OR = 1.82, 95% CI: 1.09–3.02, *p* = 0.021), and alcohol use (OR = 1.43, 95% CI: 1.02–2.01, *p* = 0.038). These variables were included in the multivariate logistic regression model, where corticosteroid use (OR = 1.84, 95% CI: 1.27–2.71, *p* = 0.002), low BMD (OR = 1.62, 95% CI: 1.15–2.37, *p* = 0.008), and advanced age ( ≥ 75 years) (OR = 1.49, 95% CI: 1.02–2.22, *p* = 0.042) remained significant independent risk factors for AVNFH. Smoking, hypertension, and diabetes were not statistically significant in the multivariate analysis. The results of the logistic regression models are summarized in Table 2.

**TABLE 2:** Multivariate and univariate logistic regression analysis of patients with avascular necrosis of the femoral head for identifying risk factors for disease progression.

	Univariate analysis			Multivariate analysis		
Variables	*P*	OR	95% CI	*P*	OR	95% CI
Age ≥ 75 y/< 75 y	0.018	1.58	1.08–2.31	0.042	1.49	1.02–2.22
BMI ≥ 25 / < 25	0.227	1.22	0.88–1.71	0.174	1.20	0.87–1.65
Gender (male/female)	0.541	1.12	0.77–1.63	–	–	–
Corticosteroid use (yes/no)	< 0.001	2.01	1.41–2.86	0.002	1.84	1.27–2.71
Smoking history (yes/no)	0.291	1.25	0.83–1.88	–	–	–
Alcohol use (yes/no)	0.038	1.43	1.02–2.01	0.062	1.36	0.98–1.94
BMD low/normal	0.002	1.76	1.24–2.51	0.008	1.62	1.15–2.37
Hypertension (yes/no)	0.464	1.15	0.78–1.69	–	–	–
Diabetes (yes/no)	0.379	1.18	0.82–1.72	–	–	–
Osteoporosis (yes/no)	0.071	1.35	0.97–1.89	0.098	1.31	0.95–1.84
Femoral fracture (yes/no)	0.021	1.82	1.09–3.02	0.057	1.59	0.98–2.59
MRI abnormality (yes/no)	0.108	1.31	0.94–1.84	–	–	–
Cholesterol > 5.2 mmol/L	0.156	1.28	0.91–1.81	–	–	–
LDL-C > 3.4 mmol/L	0.193	1.25	0.89–1.76	–	–	–
Triglyceride > 1.7 mmol/L	0.482	1.12	0.81–1.56	–	–	–
Vitamin D deficiency (yes/no)	0.091	1.33	0.95–1.86	0.119	1.28	0.93–1.77
Family history (yes/no)	0.341	1.20	0.82–1.76	–	–	–

OR, Odds Ratio; CI, Confidence Interval; BMD, Bone Mineral Density; LDL-C, Low-Density Lipoprotein Cholesterol. Variables with *p* < 0.1 in univariate analysis were included in the multivariate model.

### Model performance and ROC curve analysis

**FIGURE 2:**
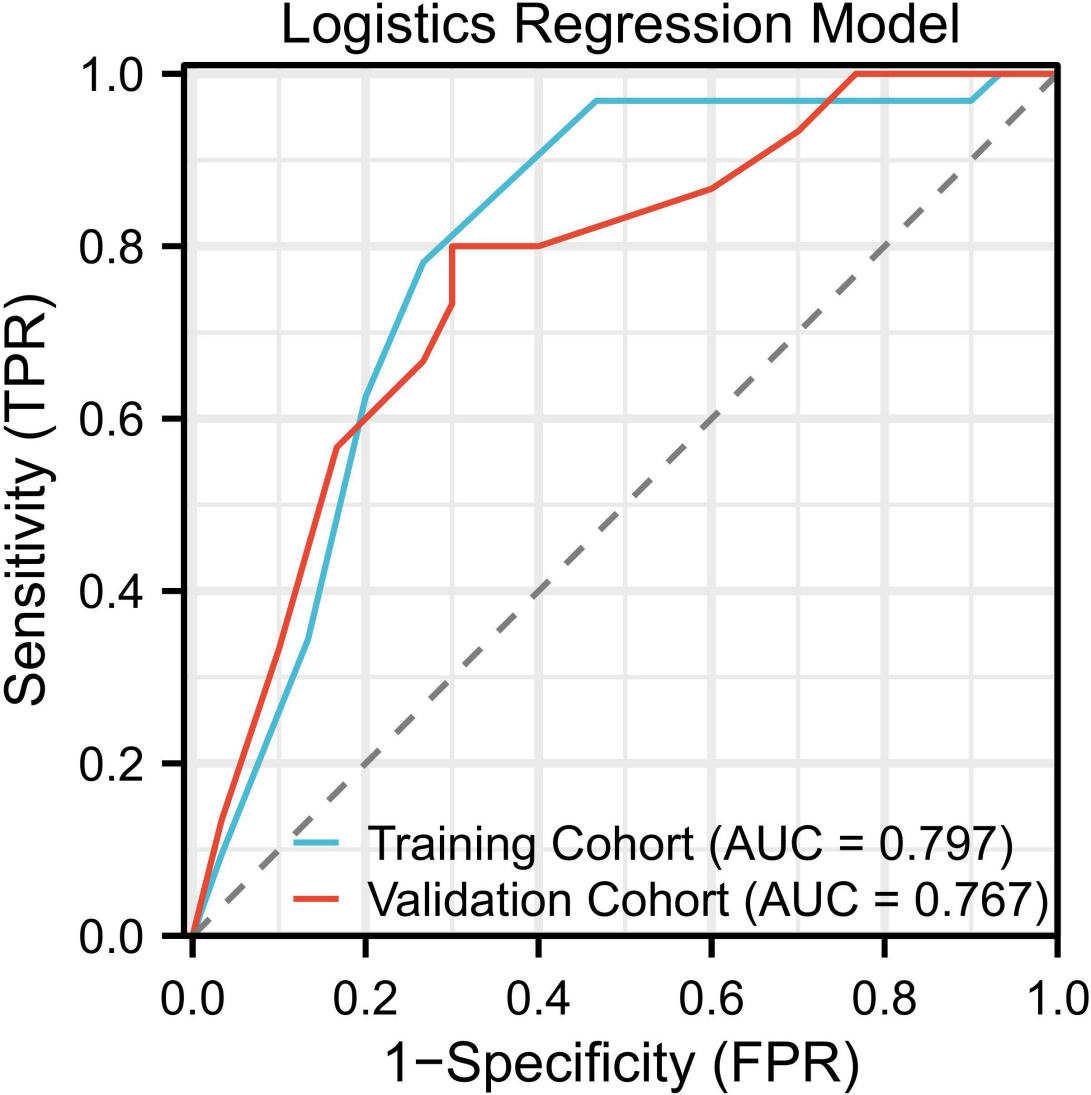
ROC curves for the logistic regression model showing AUCs of 0.797 (training) and 0.767 (validation).

The logistic regression model showed moderate predictive performance, with an area under the ROC curve (AUC) of 0.797 in the training cohort and 0.767 in the validation cohort. In the validation cohort, the model achieved a sensitivity of 71.3% and a specificity of 76.9%, indicating a balanced yet limited discriminative capability. The ROC curves for both training and validation sets are illustrated in Figure 2.

### Feature importance in the random forest model

**FIGURE 3:**
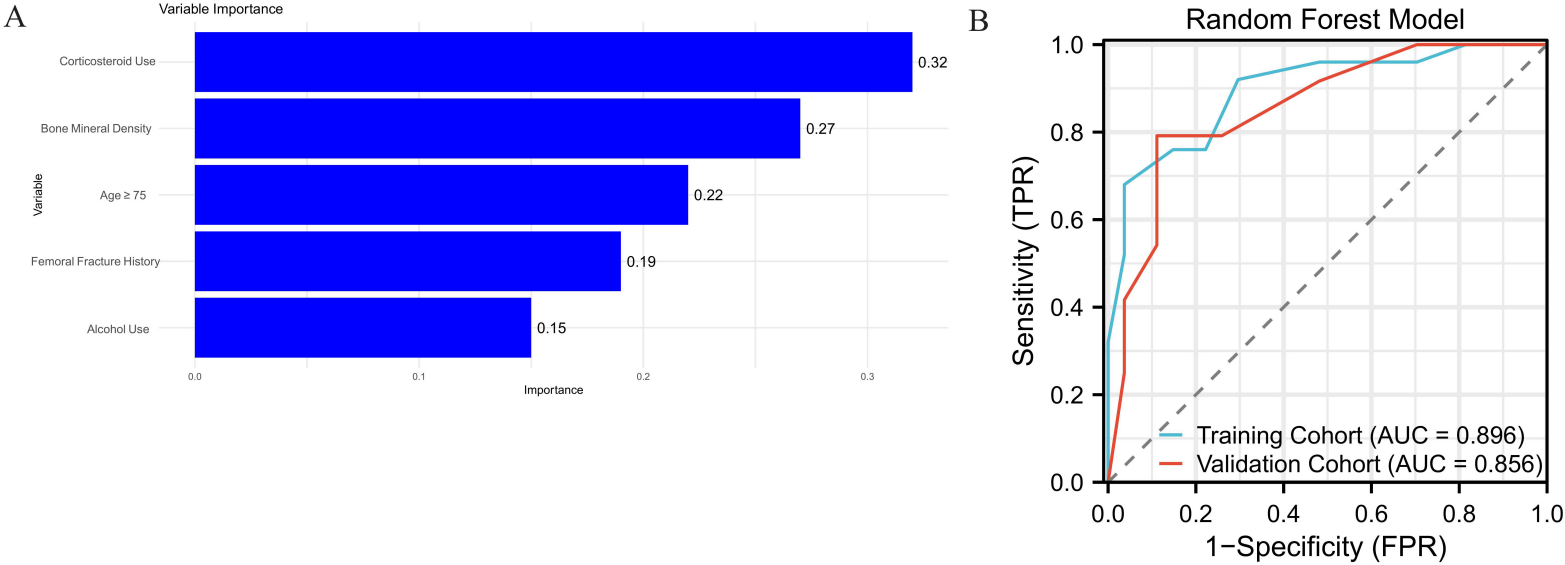
(A) Top five most important variables in the Random Forest model. (B) ROC curves for the Random Forest model with AUCs of 0.896 (training) and 0.856 (validation).

Feature importance analysis from the Random Forest model identified corticosteroid use as the most influential variable, followed by low bone mineral density, age ≥ 75 years, history of femoral fracture, and alcohol use. These five variables had the highest contribution to the model’s classification decisions, with normalized importance scores of 0.32, 0.27, 0.22, 0.19, and 0.15, respectively. The relative importance of these features is presented in Figure 3A.

In comparison, the Random Forest model demonstrated better overall predictive power. It achieved an AUC of 0.896 in the training cohort, along with a sensitivity of 82.4%, specificity of 84.3%, and an overall accuracy of 83.5%. In the validation cohort, the AUC was 0.856, with a sensitivity of 79.2%, specificity of 82.1%, and overall accuracy of 80.4%. These results suggest that the Random Forest model provided improved generalizability and robustness in classifying patients with avascular necrosis of the femoral head. The corresponding ROC curves are shown in Figure 3B. Supplementary Figure 1 shows the decision curve analysis (DCA) of the random forest model and the logistics regression model, suggesting that the RF model has higher benefits.

### Comparison between logistic regression and random forest models

The performance metrics for both models (Logistic Regression and Random Forest) are shown in Table 3. The Random Forest (RF) model consistently outperformed the Logistic Regression (LR) model across all evaluation metrics in both the training and validation sets. Specifically, the RF model achieved an accuracy of 84.6% (95% CI: 79.5–89.6%) in the training cohort and 83.2% (95% CI: 74.9–89.3%) in the validation cohort, with a sensitivity of 82.5% (95% CI: 77.0–87.5%) and specificity of 87.0% (95% CI: 81.4–91.6%). In comparison, the LR model achieved an accuracy of 81.2% (95% CI: 78.2–84.2%) in the training cohort and 79.0% (95% CI: 74.9–82.9%) in the validation cohort, with a sensitivity of 72.0% (95% CI: 66.0–78.0%) and specificity of 83.0% (95% CI: 79.3–86.7%). Moreover, the RF model demonstrated a higher F1-score (0.855 vs. 0.776 in the training set; 0.849 vs. 0.759 in the validation set), indicating a better balance between precision and recall. These results, along with the 95% confidence intervals, indicate that the RF model provides more reliable and stable predictions across both the training and validation datasets compared to the Logistic Regression model. In addition to accuracy, sensitivity, specificity, and F1 score, the predictive positive value (PPV, or precision) was calculated for both models. The Logistic Regression model achieved a PPV of 0.760 (95% CI: 0.715–0.805) in the training cohort and 0.740 (95% CI: 0.692–0.787) in the validation cohort. The Random Forest model achieved a PPV of 0.830 (95% CI: 0.791–0.860) in the training cohort and 0.815 (95% CI: 0.770–0.845) in the validation cohort. Overall, the Random Forest model demonstrated greater generalizability, robustness, and clinical interpretability than the logistic regression model, suggesting its superior suitability for identifying high-risk elderly patients with AVNFH.

**TABLE 3:** Evaluation indicators for each model.

Model	Accuracy	Sensitivity	Specificity	Precision	Recall	F1 score	PPV	ROC-AUC	95% CI
Logistic regression (training)	0.812	0.720	0.830	0.760	0.721	0.776	0.760	0.797	0.733–0.875
Validation	0.790	0.688	0.818	0.740	0.692	0.759	0.740	0.767	0.701–0.847
Random Forest (training)	0.846	0.825	0.870	0.830	0.824	0.855	0.830	0.896	0.795–0.976
Validation	0.832	0.811	0.860	0.815	0.808	0.849	0.815	0.856	0.749–0.938

ROC, receiver operator characteristic; AUC, area under the curve; CI, confidence interval.

## Discussion

This study highlights the advantage of machine learning approaches—especially the Random Forest algorithm—over traditional logistic regression in forecasting AVNFH risk in the elderly. While both models achieved reasonable predictive performance, Random Forest consistently outperformed logistic regression in terms of accuracy, sensitivity, specificity, and AUC. Its superior results stem from its capacity to model complex, nonlinear associations and higher-order interactions among clinical features, which are often beyond the scope of conventional regression techniques. Furthermore, the ensemble nature of Random Forest, which integrates predictions from multiple decision trees, enhances model stability and mitigates overfitting, making it particularly effective for predicting multifactorial conditions like AVNFH (13–16).

Our results align with a growing body of literature advocating for the use of machine learning techniques in clinical prediction modeling. Studies in various domains—including cardiovascular risk, osteoarthritis progression, and postoperative complication prediction—have reported the superior performance of ensemble learning methods such as Random Forest over traditional statistical tools. For instance, Cheng et al. (17) demonstrated that machine learning models could predict osteonecrosis in patients with systemic lupus erythematosus more accurately than standard risk scores. Similarly, Kunze et al. (18). showed that Random Forest-based models were highly effective in identifying hip arthroplasty failure risk. In our study, while logistic regression was able to identify key independent risk factors such as corticosteroid use, low bone mineral density, and advanced age, it fell short in distinguishing subtle interactions and had lower generalizability compared to the Random Forest model.

Several important clinical variables were consistently associated with AVNFH in our analysis. Long-term corticosteroid use emerged as the most significant predictor, which is consistent with extensive prior research showing that glucocorticoids impair osteoblast function, promote adipogenesis, and reduce femoral head vascularity. Low bone mineral density was another prominent risk factor, likely reflecting the structural fragility of the femoral head in elderly individuals with osteoporosis. Advanced age ( ≥ 75 years) was independently associated with increased AVNFH risk, possibly due to age-related vascular insufficiency, impaired bone remodeling, and a higher burden of systemic comorbidities. History of femoral neck fracture and alcohol consumption were also significant, though to a lesser extent. These findings are consistent with existing orthopedic and geriatric literature, reinforcing the multifactorial etiology of AVNFH (19, 20). Our results have important clinical implications. Early identification of patients at high risk of AVNFH enables timely preventive strategies, such as reduction in corticosteroid exposure, closer monitoring via imaging, and initiation of bone-preserving therapies. Particularly in elderly populations, where surgical options are often complicated by comorbidities, conservative management guided by accurate risk stratification may significantly reduce the burden of advanced femoral head collapse. Moreover, the Random Forest model provides interpretable variable importance scores, which can be translated into user-friendly decision-support tools in clinical settings. However, several potential biases and confounding factors could affect the performance of our models. First, the retrospective nature of the study may introduce selection bias, as patients with severe comorbidities or specific treatment histories may have been underrepresented. Additionally, the homogeneity of the sample from a single center limits the generalizability of our findings. We also acknowledge that unmeasured factors, such as genetic predisposition or lifestyle variables, could confound the observed relationships. To mitigate these issues, future studies could include a multicenter, prospective cohort with more diverse patient populations and a broader range of clinical data. Furthermore, we recommend integrating additional features, such as genetic markers or physical activity data, to capture a more comprehensive risk profile.

Our study has several limitations. First, it was conducted at a single center with a relatively homogeneous patient population, which may limit the generalizability of the findings. To address this, we recommend conducting external validation using larger, multicenter cohorts to evaluate the model’s performance in diverse clinical settings. Additionally, the sample size of this study was relatively small, which may affect the statistical power of the results. Future studies should aim to validate these findings in larger, more diverse populations to enhance generalizability. Secondly, the exclusion of certain variables, such as genetic predisposition, lifestyle factors, and other potential confounders, may have limited the comprehensiveness of the model. Incorporating these factors could improve the predictive accuracy and provide a more holistic risk assessment. Furthermore, while the Random Forest (RF) model demonstrated high predictive performance, it remains a “black box” compared to traditional models, with limited interpretability. To address this limitation, we propose integrating explainable artificial intelligence (XAI) tools, such as SHAP (Shapley Additive Explanations) or LIME (Local Interpretable Model-Agnostic Explanations), in future studies. These techniques would help elucidate individual-level predictions and enhance transparency, thereby fostering greater trust in the model’s decision-making process among clinicians (21, 22).

In our study, the RF model outperformed logistic regression in both the training and validation cohorts. One potential explanation is the RF model’s ability to capture complex non-linear relationships and high-order interactions among features, which may be particularly relevant in multifactorial conditions such as avascular necrosis of the femoral head (AVNFH). To explore the interpretability of the RF model, we conducted a feature importance analysis based on the Gini index, as presented in Figure 3A. Corticosteroid use, bone mineral density, and advanced age emerged as the top three predictors, which is consistent with well-established clinical risk factors for AVNFH. In particular, corticosteroid use has been widely implicated in disrupting bone microvascularity and metabolism, leading to ischemic changes in the femoral head. The inclusion of fracture history and alcohol use among the top-ranked variables further supports the clinical validity of the model’s decision process.

Future directions should also explore the integration of multimodal data sources—including radiomics features from MRI, serum biomarkers, and electronic health record (EHR) streams—to improve prediction accuracy and facilitate real-time clinical application. Additionally, prospective studies involving real-world implementation and impact assessment of machine learning-based risk prediction tools are needed to validate their clinical utility. While this study was based on a single-center cohort, we recognize the value of external validation using established publicly available datasets. In future work, we plan to validate our model using well-known datasets to assess its performance across different populations and further improve its accuracy.

## Conclusion

This study demonstrates that machine learning models, especially the Random Forest algorithm, provide superior predictive performance compared to traditional logistic regression in identifying elderly patients at risk for avascular necrosis of the femoral head. The Random Forest model’s ability to capture complex variable interactions and its robustness across training and validation sets underline its value as a decision-support tool in orthopedic practice. Key clinical factors, including corticosteroid use, bone mineral density, and advanced age, were identified as significant predictors and may guide targeted monitoring and preventive interventions. Future research should aim to externally validate these findings, incorporate additional risk variables, and explore interpretable machine learning frameworks to promote clinical integration. These insights have the potential to enhance early identification, reduce surgical burden, and improve patient outcomes in geriatric populations at risk of femoral head necrosis.

## Data Availability

The original contributions presented in the study are included in the article/Supplementary material, further inquiries can be directed to the corresponding author.

## Supplementary material

Supplementary Figure 1 Decision curve analysis (DCA) for the Random Forest model and logistics regression model.
