# Supplementary figures and images for: Enhanced risk prediction of femoral head osteonecrosis in the elderly: a comparative study of random forest and logistic regression models
Source: Front Med (Lausanne). 2025 Dec 10;12:1640085. doi: 10.3389/fmed.2025.1640085 (PMC12728075; doi:10.3389/fmed.2025.1640085)

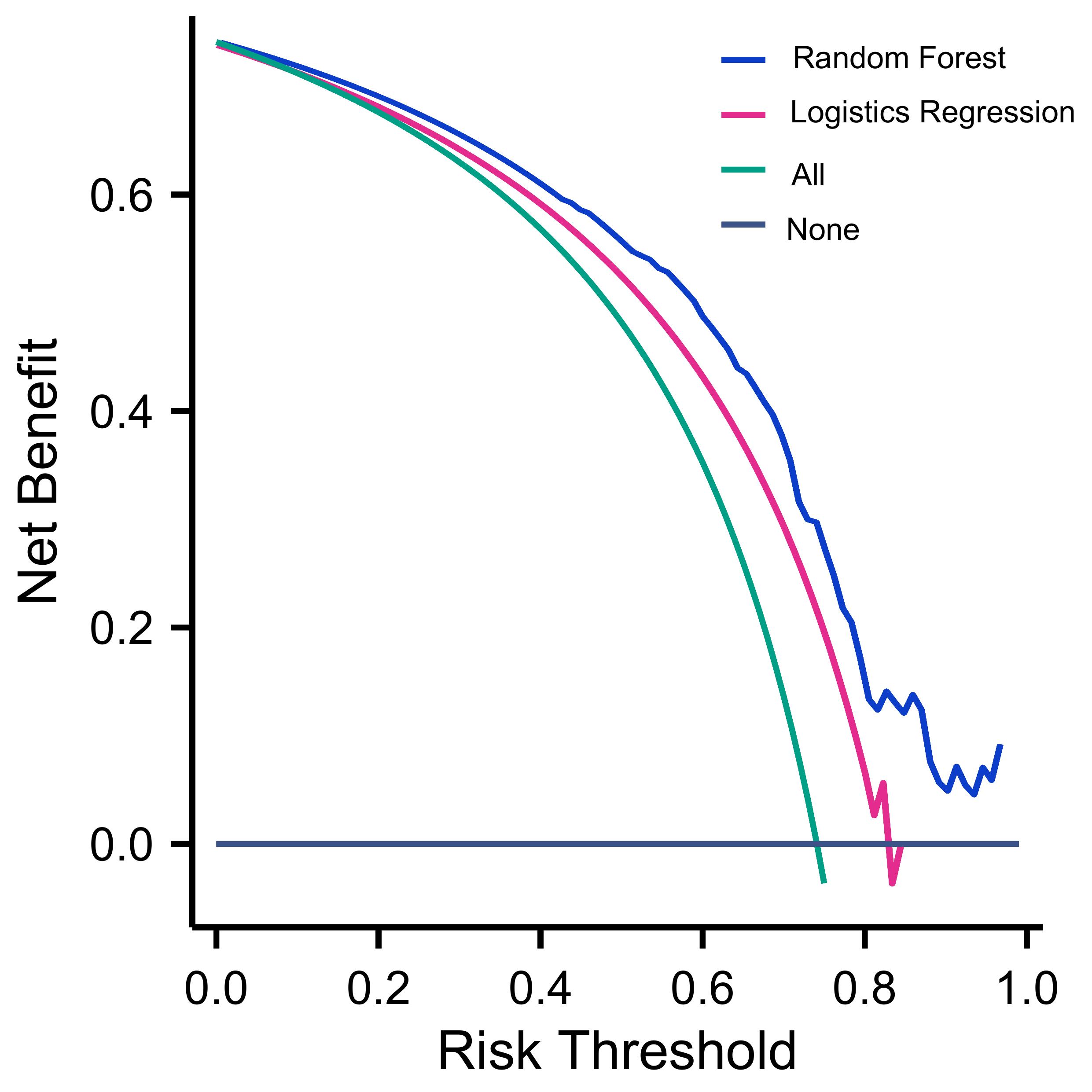

Supplement: Supplementary Figure 1 — Decision curve analysis (DCA) for the Random Forest model and logistics regression model. [file Image_1.jpeg]
